# Fall risk factors among poly-medicated older Lebanese patients in primary care settings: a secondary cross-sectional analysis of the “MGPIDP-L project”

**DOI:** 10.1186/s12877-024-04951-0

**Published:** 2024-04-10

**Authors:** Sarah El Khatib, Carmela Bou Malham, Sandrine Andrieu, Mathilde Strumia, Philippe Cestac, Pascale Salameh

**Affiliations:** 1 Paul Sabatier University III, 31063 Toulouse, France; 2 Aging and Research Team, Center for Epidemiology and Research in POPulation Health (CERPOP), Toulouse University, Inserm, Paul Sabatier University, Faculty of Medecine, 37 Allées J.Guesde, 31000 Toulouse, France; 3 Department of Pharmacy, Toulouse University Hospitals, Purpan Hospital, 31059 Toulouse, France; 4 School of Medicine, Lebanese American University, Byblos, 1401 Lebanon; 5 University of Nicosia Medical School, 1065 Nicosia, Cyprus; 6 Faculty of Pharmacy, Lebanese University, Hadath, 1100 Lebanon; 7 Institut National de Santé Publique, Epidémiologie Clinique Et Toxicologie INSPECT-LB), Beirut, 1100 Lebanon

**Keywords:** Inappropriate drug use, Fall risk increasing drugs, Primary care, Older adults

## Abstract

**Background:**

Falling is a major concern for the health of older adults and significantly affects their quality of life. Identifying the various risk factors and the differences between older patients can be challenging. The objective of this study was to identify the risk factors for falls among polymedicated community-dwelling older Lebanese patients following a medication review.

**Methods:**

In this analytical cross-sectional study, we examined the risk factors for falls in 850 patients aged ≥ 65 years who were taking ≥ 5 medications daily. The study involved conducting a medication review over the course of a year in primary care settings and using multivariate logistic regression analysis to analyze the data.

**Results:**

Our results showed that 106 (19.5%) of the 850 included patients had fallen at least once in the three months prior to the medication review. Loss of appetite and functional dependence were identified as the most significant predictors of falls ORa = 3.020, CI [2.074–4.397] and ORa = 2.877, CI [1.787–4.632], respectively. Other risk factors for falls included drowsiness ORa = 2.172, CI [1.499–3.145], and the use of beta-blockers ORa = 1.943, CI [1.339–2.820].

**Conclusion:**

Our study highlights the importance of addressing multiple risk factors for falls among Lebanese older adults and emphasizes the need for customized interventions and ongoing monitoring to prevent falls and improve health outcomes. This study sheds light on a critical issue in the Lebanese older population and provides valuable insight into the complex nature of falls among poly-medicated Lebanese community-dwelling older adults.

**Trial registration:**

2021REC-001- INSPECT -09–04.

## Background

Given the ageing of societies, falls among community-dwelling older adults are considered a major public health problem that needs to be addressed due to its clinical and socioeconomic implications [1].

The number of older adults in the Arab world is expected to double by 2050 and treble by 2100, with disability, functional dependence, and chronic noncommunicable diseases being the main causes of morbidity and mortality [2]. The published literature describes a high worldwide prevalence of falls (26.5%) among older adults [3]. Moreover, in Arab countries, the reported rates vary, with instances of 21.9% in Lebanon [4], 34% in Qatar [5], and a peak of 57.7% in Saudi Arabia [6]. Given the elevated prevalence rates, the World Falls guidelines advocate for the integration of fall screening into the routine comprehensive geriatric assessment of community-dwelling older adult [7].

Multiple predisposing factors put community-dwelling older adults at higher risk for falls. These include sociodemographic characteristics [8–10], geriatric syndromes [11, 12], clinical, and medical conditions, among others [13]. Fall risk increases linearly with the number of these factors [14].

Furthermore, older patients often suffer from the existence of several comorbidities leading to the concomitant use of multiple drugs. The pharmacodynamic and pharmacokinetic properties of drugs also change with age, due to decreased renal and hepatic function, altered fat distribution, and reduced metabolic rate. This results in a different response to drug therapy, rendering older adults more susceptible to adverse drug events (ADEs) if these changes are not taken into account [15–19]. Several studies have evaluated the association between medication use and the increased likelihood of fall [13, 20]. Three systematic reviews and meta-analyses conducted by Seppala et al. highlighted the occurrence of falls associated with particular classes of medications such as cardiovascular drugs, psychotropic drugs, opioids, and proton pump inhibitors [13, 20–23].

To limit ADEs associated with falls in older adults, the Swedish National Board of Health has classified certain medications as ‘fall risk-increasing drugs’ (FRIDs) [24]. Screening of all these factors including FRIDs through a medication review may be a practical way to prevent future falls.

A well-structured medication review in older patients not only leads to the optimization of medical prescriptions but also allows the pharmacist to determine what diseases the patient is suffering from, and to evaluate the adherence and management of their treatment. A thorough analysis of all these elements will make it possible to prevent further deterioration of the patient’s health and ensure his or her well-being [25–28].

In Lebanon, no previous study has evaluated the risk of falls among community-dwelling older adults and its associated factors. One study conducted at a tertiary care center in Lebanon showed that 99.2% of falls occurred at home and emphasized the importance of public awareness and the necessity of screening for fall risk factors [29].

This study aimed to look at the risk factors associated with falls in community-dwelling older Lebanese patients taking more than 5 different medications daily [30] through a medication review to address the problem of falls in older adults.

## Methods

### Study design and setting

This study is a secondary analytical cross-sectional study, designed to examine falls and their associated risk factors following a medication review. This study is part of the project entitled “Management of potentially inappropriate drug prescribing among Lebanese patients in primary care settings” (MGPIDP-L). The MGPIDP-L project aimed to assess potentially inappropriate drug prescribing (PIDPs) in Lebanese older patients in primary care settings (upon hospital discharge, at the patient’s home, and in community pharmacies).

Participants: Patients were systematically recruited over a 12-month period from a wide range of primary care settings, such as hospitals upon discharge, through home visits, and within community pharmacies. Contact was established with community and hospital pharmacies in Lebanon through conferences, phone calls, and mails to explain the study's rationale and methodology. Hospital pharmacists selected recently discharged patients meeting inclusion criteria, while community pharmacists displayed posters to encourage patient’s participation. Patients aged 65 years or older who were taking 5 or more medications per day, and who could provide all necessary medical information directly or with the assistance of a caregiver or family member in certain cases such as dementia were included. Patients not fulfilling the mentioned criteria were excluded.

During this project, a Medication review was conducted by two experienced clinical pharmacists over 12 months. Following the patient’s interview, an analysis of the patient’s medical treatment was performed. The analysis included the list of medications incorporating both those prescribed by the physician for the patient and any over-the-counter medications self-administered by the patient. Then, a formulation of justified pharmaceutical interventions was proposed for the patient’s general practitioner. After the physician’s approval, treatment modifications were communicated to the patient [28].

In the Original MGPIDP-L project, a sample size was calculated using Epi Info 7.2.5.0. For a total population size of around 600,000 Lebanese older adults, a confidence level of 99.9%, and a prevalence of 60% of inappropriate prescribing among the elderly population [31], 1083 patients were needed. Out of 1,038 patients involved in the study, 188 (18.11%) records were excluded due to incomplete, insufficient, or conflicting data. This left a sample of 850 patients enrolled with complete data during the 12-month inclusion period. In this study, we used this convenience sample for secondary analysis. Based on the following calculation: Using the G-Power software, version 3.0.10, this sample was sufficient to detect a calculated difference in fall rate of 0.0526, an alpha error of 5%, and a power of 80%. Given the expected fall rate of 0.05, 25 predictors could be included in the model.

### Tool used and analysis performed

The data collected directly from the patients included information on patients’ sociodemographic characteristics, chronic diseases, presence of geriatric syndromes including previous falls, medical treatment, and its management, tolerability, and compliance. The collected data were analyzed using a combination of both implicit and explicit approaches. The implicit criteria are based on the patient's global assessment while explicit criteria consist of predefined indicators of potentially inappropriate medication use in the geriatric population, established by expert consensus. To enhance the detection of potentially inappropriate drug prescriptions (PIDPs) and optimize medical prescribing, we utilized five distinct lists—Beers criteria, EU7-PIM, Laroche, STOPP/START, and STOPP/Frail [31–35]. Given the variation in PIDP prevalence based on the criteria employed and considering the diverse origins of medications in the Lebanese market, we combined these lists to increase the likelihood of PIDP detection. Two pharmacists assessed each medication taken by the patient to determine if it matched any of the criteria listed in the five sets. They also evaluated the appropriateness of each medication based on the patient's clinical condition and determined whether a pharmaceutical intervention should be recommended to the general practitioner. In the current study, the medical prescriptions of the included subjects were also analyzed to detect the presence of FRIDs according to the Swedish National Board of Health. The Swedish National Board of Health and Welfare defined FRID classes and further classified them into drug categories that cause a high risk of falling, orthostatic hypotension, and/or systemic hypotension [24].

### Assessment and predictors of falls

The variable of interest “the previous fall” was self-reported by the study participants. In the questionnaire, participants were asked whether they had fallen at least once in the past 3 months. The period of 3 months was chosen to avoid bias due to memory loss and to associate the fall with medication use recorded at baseline [36, 37]. These subjects were compared with participants who had not fallen to identify variables associated with increased fall risk.

Potential risk factors for falls were divided into 3 categories:

- Sociodemographic variables

Age (young old (65–74); middle-old (75–84); old-old (≥ 85)), biological gender, weight, living arrangement (patient lives alone, with a spouse, or with family), residence area (North Lebanon, Beirut, Mount Lebanon, Beqaa and South), and type of assistance needed (nurse, housekeeper, physical therapy). The choice of these forms of assistance was influenced by the Lebanese context, where hiring a housekeeper was feasible for many individuals prior to the economic downturn. This served as a significant alternative to nursing homes, particularly for families with members who go out to work or are residing abroad. Registered nurses are typically required for older adults with specific healthcare needs. Additionally, in instances of falls, patients needing specialized medical attention are often hospitalized in tertiary care facilities due to the increased expenses associated with home-based nursing care and rehabilitation.

- Disease-related variables

Existing medical conditions and/or geriatric syndromes [38] (Frailty, urine incontinence, insomnia, daytime drowsiness, involuntary weight loss, loss of appetite).

- Medication-related variables

Total number of PIDPs/medical prescriptions, presence of fall risk-increasing drugs (FRIDs)/medical prescriptions, and FRID classes used according to the Swedish National Board of Health [24], dichotomous questions related to the patient’s behavior toward his medical treatment. These questions covered various aspects such as medical treatment management (e.g., pill organizer usage, medication pick up, self-dependent medication management), prescription of drugs (e.g., perception of medication quantity, adaptability to lifestyle), stock management (e.g., medication availability, excess, sharing), preparation and administration challenges, medication utility, side effects, treatment follow up, and self-medication tendencies.

### Statistical analysis

SPSS version 26 was used to analyze the data. The dependent variable was the self-reported fall history (fallers vs. non-fallers). The chi-square test was used to assess bivariate associations between categorical variables and fall history. The student-independent t-test and Wilcoxon-Mann–Whitney tests were used when appropriate to compare means between fallers and non-fallers for continuous variables (weight, total number of medications, total number of diseases, total number of identified (PIDPs)/prescriptions, and total number of (FRIDs)/prescription) after checking for normality using the Kolmogorov–Smirnov test. Given the exploratory nature of our analysis, we opted not to adjust for multiple testing in the bivariate analyses as an initial step to identify variables that might warrant further investigation in subsequent multivariate analyses.

Multivariate logistic regression analysis was performed to identify independent factors associated with a fall in the past 3 months using a backward likelihood ratio method. Adjusted odds ratios (OR) with 95% confidence intervals (CI) were calculated. To mitigate the risk of chance findings due to multiple tests, we applied the Bonferroni correction which adjusts the significance threshold for each test to maintain an overall experiment-wise error rate. The Hosmer & Lemeshow test was used to ensure sample adequacy. The evaluation of collinearity in our analysis was conducted by computing Variance Inflation Factors (VIF) for each predictor variable incorporated in the different models. The main explanatory variables that were associated with fall in the bivariate analysis (*p* < 0.05) were included in the models to decrease potential confounding. Considering the case-to-independent variables (IVs) ratio of 40:1 [39], three consecutive regression models were conducted. Sociodemographic and clinical predictors variables from Table 1 were included in the first model. Medication-related variables from Tables 2, and 3 were introduced into the second model. The final model (model 3) was run with significantly associated variables from the previous two models as explanatory variables. The total number of drugs listed as FRID /prescription and the total number of PIDPs/prescription were also forced. In all cases, a *P*-value of less than 0.05, adjusted for the number of tests using the Bonferroni correction, was considered significant. This adjustment ensured a more conservative interpretation of statistical significance, guarding against inflated Type I error rates associated with multiple comparisons.

**Table 1:** Sociodemographic and clinical characteristics

	**Variables**		**Fall in the past 3 months n (%)**		***P*-value**
			**Yes(*N* = 166) n (%)**	**No (*N* = 684) n (%)**	
**Socio-demographic characteristics**	Gender	Female	92(55.4)	339(49.6)	0.175^1^
	Age group	65–74	72(43.4)	336(49.1)	0.395^1^
		75–84	62(37.3)	235(34.4)	
		≥ 85	32(19.3)	113(16.5)	
	Weight (Mean (SD)		73.23(12.43)	76.95 (12.68)	0.000^*2^
	Region	Beirut	35(21.1)	108(15.8)	0.050^1^
		Mount Lebanon	56(33.7)	267(39)	
		South	15(9.0)	108(15.8)	
		Beqaa	22(13.3)	78(11.4)	
		North	38(22.9)	123(18)	
	Living arrangement	Alone	16(9.6)	84(12.3)	0.204^1^
		With spouse	50(30.1)	163(23.8)	
		With family	100(60.2)	437(63.9)	
	Assistance	Housekeeper	44(26.5)	132(19.3)	0.040^*1^
		Registered Nurse	11(6.6)	21(3.1)	0.031^*1^
		Physiotherapist	13(7.8)	19(2.8)	0.020^*1^
**Geriatric syndromes**	Assessment of Frailty level according to Find auto- questionnaire	Robust	44(26.5)	305(44.6)	< 0.001^*1^
		Disabled/Dependent	78(47)	181(26.5)	
		Fragile	44(26.5)	198(28.9)	
	Insomnia		102(61.4)	257(37.5)	< 0.001^*1^
	Daytime drowsiness		112(67.5)	311(45.5)	< 0.001^*1^
	Weight loss > 4.5 kg/year		66(39.8)	139(20.3)	< 0.001^*1^
	Loss of appetite		80(48.2)	127(18.5)	< 0.001^*1^
	Urinary incontinence		58(35.2)	142(20.7)	< 0.001^*1^
**Comorbidities**	Alzheimer/dementia		11(6.6)	10(1.5)	0.001^*3^
	Atrial Fibrillation		21(12.7)	110(16.1)	0.284 ^1^
	Cancer		6(3.6)	10(1.5)	0.102^3^
	Depression		26(15.7)	67(9.8)	0.030 ^*1^
	Diabetes		73(44.0)	281(41.1)	0.497 ^1^
	Dyslipidemia		70(42.2)	345(50.4)	0.056 ^1^
	Epilepsy		8(4.8)	11(1.6)	0.019 ^*3^
	Heart Failure		23(13.9)	87(12.7)	0.696 ^1^
	Hypertension		129(77.8)	548(80.11)	0.490 ^1^
	Myocardial infarction		40(24.1)	199(29.1)	0.199 ^1^
	Osteoporosis		48(28.9)	115(16.8)	< 0.001^*1^
	Parkinson		6(3.6)	10(1.5)	0.102 ^3^
	Pulmonary Disease		22(13.2)	77(11.26)	0.472 ^1^
**Number of diseases Mean (SD)**			3.54(1.55)	3.28(1.48)	0.046^2^
**Number of drugs Mean (SD)**			7.26(2.69)	7.06(2.39)	0.802^2^
**Total number of PIDPs/prescription Mean (SD)**			4.69(4.04)	4.9(3.66)	0.958^2^

^*^P < 0.05

^1^Chi-square test

^2^Mann-Whitney U test

^3^Fisher exact test

**Table 2:** Patient behavior toward his/her medical treatment

**Patient’s behavior toward his/her medical treatment**	**Fall in the past 3 months**		***P*-value**
	Yes (*N* = 166) *n* (%)	No (*N* = 684) n (%)	
**General treatment management**			
Do you use a pill organizer?	28(16.9)	85(12.4)	0.131^1^
Do you pick up your medication from the pharmacy?	53(32.1)	297(43.4)	0.008^*1^
Do you manage taking your medication on your own?	62(37.3)	365(53.4)	0.000^*1^
**Prescription of drugs**			
Is your medications’ schedule adapted to fit your lifestyle?	131(79.4)	588(86)	0.041^*1^
Do you think that you take too many drugs?	68(8)	244(28.7)	0.689
**Management of the stock of medications**			
Do you ever run out of drugs?	26(15.7)	70(10.2)	0.047^*1^
Do you have an excess of medications?	29(16.2)	96(14)	0.244
Do you share any medication with a relative?	13(7.3)	48(7.2)	0.964
**Preparation and administration of medications**			
Do you face any problem with the dosage form of some of your medications (swallow tablets, counting the drops?)	19(11.5)	48(7)	0.054^1^
Are there any medications you are crushing or capsules you are opening?	10(1.2)	26(3.1)	0.314
Do you ever forget to take your medications?	27(15.1)	111(16.6)	0.633
During the last 2 weeks, were there any days when you did not take your medication?	11(6.1)	59(8.8)	0.250
**Medications utility**			
Do you think that some of your medications are not useful	12(6.7)	44(6.6)	0.944
**Side effects**			
(Did you ever suffered from any side effects after taking a medication (s)?)	22(13.3)	63(9.2)	0.119^1^
**Treatment follow up**			
If some of your medications require constant monitoring by doing a blood test, will you have any difficulty in doing it?	17(10.2)	47(6.9)	0.14^1^
**Self-medication**			
Do you often use medications without a prescription?	22(13.3)	154(22.5)	0.08^1^
Have you ever decreased or increased the dose of certain drug on your own?)	12(6.7)	47(7)	0.888

^*^P < 0.05

^1^Chi-square test

**Table 3:** Number of drugs classified as FRIDs ^a^

**FRIDs Class**	**ATC Codes** ^b^	**Number of Drugs**		*P*-value
		**Fallers (*N* = 166) n (%)**	**Non fallers (*N* = 684) n (%)**	
**Drugs that cause high risk of falling**				
Opioids	N02A	2(1.2)	19(2.8)	0.401
Antipsychotics	N05A(excluding Lithium)	12(7.3)	24 (3.5)	0.032^*^
Anxiolytics	N05B	13(7.8)	42(6.2)	0.433
Hypnotics and sedatives	N05C	1(0.6)	3(0.4)	0.582
Antidepressants	N06A	26(15.7)	63(9.2)	0.015^*^
**Drugs that cause orthostatism/hypotension**				
Vasodilators used in cardiac diseases	C01D	6(3.6)	67(9.8)	0.011^*^
Anti-adrenergic-Antihypertensive	C02	13(7.8)	22(3.2)	0.007^*^
Diuretics	C03	72(43.4)	248(36.2)	0.087
Beta-blockers	C07	102(61.4)	350(51.1)	0.017^*^
Calcium channel blockers	C08	57(34.3)	193(28.3)	0.126
Agents acting on the renin-angiotensin system	C09	76(45.8)	319(46.7)	0.831
Alpha-adrenoreceptor antagonists	G04CA	13(7.8)	55(8.1)	0.925
Anti-Parkinson drugs	N04B	3(1.8)	18(2.6)	0.781
**Existence of a least one Medication classified as FRID**		145(87.3)	618(90.4)	0.252^1^
**Total number of FRIDs medication /prescription (Mean (SD)**		2.23(1.55)	2.3(1.44)	0.338^2^

^*^P < 0.05

^a^ Fall risk increasing drugs classified according to the Swedish National Board of Health and Welfare [24]

^b^ Anatomical Therapeutic Chemical

## Results

### Socio-demographic characteristics

In total, four hospitals (located in Beirut, Mount Lebanon, North and South) and ten community pharmacies (two in each region) participated in the study. 850 patients were included from the MGPIDP-L project. Of those, 166 (19.5%) had experienced a recent fall in the past three months. No difference between fallers and non-fallers was found in terms of age, gender, living arrangement, and residence area in our sample. However, fallers relied significantly more on different types of assistance when compared to non-fallers: 26.5%(*n* = 44) of them were helped by a housekeeper versus (vs.) 19.3%(*n* = 132) of non-fallers, 6.6% (*n* = 11) needed assistance of a registered nurse vs. 3.3% (*n* = 21) of non-fallers, and 7.8%(*n* = 13) necessitated physiotherapy sessions compared to 2.8%(*n* = 19) of non-fallers, Table 1.

### Disease-related factors

Geriatric syndromes were more prevalent among fallers. 26.5% (*n* = 44) were frail and 47%(78) were dependent according to the Find survey [40]. Insomnia had a high overall prevalence in fallers, thus leading to experiencing more daytime drowsiness. 61.4%(*n* = 102) and 67.5% (*n* = 112) experienced insomnia and daytime drowsiness compared to 37.5%(*n* = 257) and 45.5% (*n* = 311) in non-fallers. Urinary incontinence was also more common among fallers (35.2%(*n* = 58) vs. 20.7%(*n* = 142)). Loss of appetite was more frequent in fallers (48.2%(*n* = 80) vs.18.5%(*n* = 127)) as well as weight loss (39.8%(*n* = 66) vs. 20.3%(*n* = 139)), Table 1.

Our results showed that fallers suffered from a higher number of diseases than non-fallers. The most frequent diseases among fallers and non-fallers were hypertension, followed by diabetes and dyslipidemia. Osteoporosis (29.8% (*n* = 48). vs. 16.8%(*n* = 115)), depression (15.7%(*n* = 26) vs. 9.8%(*n* = 67)), Alzheimer’s disease or dementia (6.6% (*n* = 11) vs. 1.5%(*n* = 10)), and epilepsy (4.8%(*n* = 8) vs. 1.6%(*n* = 11)) were significantly higher in fallers than non-fallers, Table 1.

### Medication-related variables

#### Patient’s medical treatment management

Fallers depended more on assistance to take their own medical treatment (63%(*n* = 104) of fallers vs. 47%(*n* = 319) of non-fallers). 68% (*n* = 113) of them needed assistance with medication collection from the pharmacy compared to 57%(*n* = 387) of non-fallers. The management of drug stock was also a problem that was more detected among fallers. 15.7%(*n* = 26) of them would run out of medications from time to time vs. 10.2% (*n* = 70) in non-fallers. 21% (*n* = 35) of fallers felt that their medication’s schedule was not adapted to fit their lifestyle compared to 14%(*n* = 96) of non-fallers. However, self-medication was less frequent among fallers (13.3% (*n* = 22) vs. 22.5%(*n* = 154)), Table 2.

#### Analysis of medical prescriptions

In total, 396 FRIDs were identified for the 166 fallers and 1423 FRIDs for 684 non-fallers. Among drugs affecting the central nervous system, antipsychotics (7.3% (*n* = 12) vs. 3.5%(*n* = 24)) and antidepressants (15.7% (*n* = 26) vs. 9.2%(*n* = 63)) were significantly more prescribed for fallers compared to non-fallers. Since they affect the central nervous system, they increase the chances of imbalance and falls in the participants.

In addition, drugs causing orthostatic hypotension were frequently prescribed in our sample for both groups (86.3%(*n* = 342) in fallers vs.89.38%(*n* = 1272) in non-fallers). Anti-adrenergic hypertensives (C02) (7.8% (*n* = 13) vs. 3.2%(*n* = 22)) and beta-blockers (61.4%(*n* = 102) vs. 51.1%(*n* = 350)) were significantly more used in fallers. On the contrary, vasodilators prescribed in the treatment of cardiac diseases were less frequent among fallers (3.6%(*n* = 6) in fallers vs. 9.8%(*n* = 67) in non-fallers), Table 3.

However, no significant differences were found between fallers and non-fallers in terms of the total number of medications per prescription classified as FRIDs nor in the total number of identified drug-related problems, Table 3.

### Multivariate analysis

The multivariate analysis was done in three consecutive models as described in Table 4.

**Table 4:** Logistic regression analyses of factors affecting the risk of fall -Model 1,2 and 3

**Model 1**					
**Covariate**s			**OR** **a**	**95%** **CI**	***P*-Value**
Assessment of Frailty level according to Find auto- questionnaire		(Frail vs. Robust^a^)	1.259	0.770–2.058	0.359
		(Dependent vs. Robust^a^)	3.010	1.920–4.718	< 0.001^*^
Drowsiness		(Yes vs. No^a^)	1.940	1.330–2.831	0.001^*^
Weight loss > 4.5kg		(Yes vs. No^a^)	1.639	1.077–2.493	0.021
Loss of appetite		(Yes vs. No^a^)	2.692	1.803–4.019	< 0.001^*^
Osteoporosis		(Yes vs. No^a^)	1.998	1.275–3.131	0.013
Depression		(Yes vs. No^a^)	1.716	1.025–2.872	0.040
Alzheimer/Dementia		(Yes vs. No^a^)	2.374	1.009–5.584	0.048
**Model 2**					
**Covariates**			**OR a**	**95% CI**	***P*-Value**
Do you manage taking your medication on your own?		(Yes vs. No^a^)	0.455	0.318–0.651	< 0.001^*^
Do you ever run out of drugs?		(Yes vs. No^a^)	2.297	1.398–3.773	0.001^*^
Antidepressants(N06A)		(Yes vs. No^a^)	1.909	1.166–3.126	0.010
Antihypertensives (C02)		(Yes vs. No^a^)	2.417	1.171–4.989	0.017
Beta-Blockers (C07)		(Yes vs. No^a^)	1.830	1.284–2.606	0.001*
Vasodilators used in cardiac diseases (C01D)		(Yes vs. No^a^)	0.350	0.154–0.798	0.013
**Model 3**					
**Covariates**			**OR a**	**95% CI**	***P*-Value**
Assessment of Frailty level according to Find auto- questionnaire	(Frail vs. Robust ^a^)		1.396	0.859–2.270	0.179
	(Dependent vs. Robust ^**a**^)		2.877	1.787–4.632	< 0.001*
Loss of appetite	(Yes vs. No ^a^)		3.020	2.074–4.397	< 0.001*
Drowsiness	(Yes vs. No ^a^)		2.172	1.499–3.145	< 0.001*
Beta-Blockers (C07)	(Yes vs. No ^a^)		1.943	1.339–2.820	< 0.001*
Do you ever run out of drugs?	(Yes vs. No ^a^)		1.800	1.070–2.0288	0.027
Do you manage taking your medication on your own?	(Yes vs. No ^a^)		0.631	0.423–0.942	0.024

Variable(s) entered in step 1: weight, housekeeper, registered nurse, physiotherapist, find scale, insomnia, drowsiness, weight loss, loss of appetite, urinary incontinence, depression, osteoporosis, Alzheimer/dementia, epilepsy, total number of diseases

Variable(s) entered in step 1: Drug supply, medication management, medication adaptation, running out of medication. C02, N05A, N06A, C07, C01D

Variable(s) entered in step 1: medication management, running out of medication, C07, find scale, drowsiness, loss of appetite, total number of drugs listed as FRID /prescription, total number of PIDPs/prescription

^*^P < 0.0033, ^*^ *P* < 0.0055

^a^ Reference Group

In the first model, statistically significant predictors for sociodemographic and disease-related factors with a corrected Bonferroni *p*-value of 0.0033 were: functional dependence, loss of appetite, and drowsiness.

In the second model, the use of beta-blockers increased the risk of falls. Self-dependent medication management was negatively associated with falls. In contrast, patients who ran out of medication from time to time were more likely to fall. The adjusted P-value after the Bonferroni correction for model 2 was 0.0055.

The final model (Model 3) showed that having experienced a fall in the last three months was significantly associated with several risk factors considering an adjusted Bonferroni P-value of 0.0055. Loss of appetite increased the odds of falling by 3 (ORa = 3.020, CI [2.074–4.397]). The risk of fall almost tripled for dependent subjects (ORa = 2.877, CI [1.787–4.632]) and, it was doubled for patients suffering from drowsiness (ORa = 2.093, CI [1.440–3.043]). Among the FRIDs, using beta-blockers increased the odds of falling by 1.9 (ORa = 1.943, CI [1.339–2.820]), Table 4.

## Discussion

In our study population, 19.5% of the patients had experienced a fall in the three months prior to the medication review. The study found that loss of appetite and dependency were significant predictors of falls. Drowsiness was also identified as risk factor. Beta-blocker use predisposed to falls in the last three months.

Our study showed that loss of appetite is the most significant predictor of falls among Lebanese older adults. Poor appetite has been reported in the literature as a predictor of malnutrition. Nutritional status in older adults is a key predictor of both frailty and sarcopenia [41–43]. The decrease in daily energy intake leads to weight loss, disproportionate loss of lean body tissue, and reduced muscle strength. These outcomes render older adults at higher risk of imbalances and falls [44]. Recently published studies discuss a possible association between polypharmacy, weight loss, and malnutrition [45–49]. This could be explained by the fact that certain drugs have been proven to affect taste, smell, or salivation, which may lead patients to change their patterns of food or fluid intake [50]. In our study, medications causing taste disturbances were the most prevalent in patients who experienced falls such as diuretics, antidepressants, and antipsychotics, Table 3 [51]. Early detection of the loss of appetite can help to prevent falls and improve overall health outcomes in older adults. These findings highlight the importance of addressing the loss of appetite as a critical risk factor for falls in the Lebanese elderly population.

In the context of our study's findings, the observed two-fold higher risk of falls in dependent individuals reaching the stage of disabled frailty aligns with the established understanding that frailty as a clinical geriatric syndrome [52, 53] contributes significantly to vulnerability in the elderly. This vulnerability, in turn, heightens the risk of adverse outcomes including dependency, disability, falls, and increased mortality [54]. Notably, our results emphasize the importance of considering the specific disability associated with frailty as a crucial factor influencing the risk of falls among older adults.

While previous literature has consistently highlighted that frail older adults exhibit the highest risk of falls, followed by prefrail older adults [55], our study provides additional nuance by indicating that the disability aspect of frailty appears to play a pivotal role in determining the risk of falls. This nuanced understanding contributes valuable insights into the intricate relationship between frailty, disability, and the propensity for falls among the elderly population.

As demonstrated in previous findings [56], drowsiness was also among the geriatric syndromes that were found to be associated with falls. This result can be explained by the fact that there’s a common use of medications that cause sleep disturbances among fallers [31, 57–61]. Drugs that affect the brain (antidepressants and antipsychotics) and some antihypertensive agents (adrenergic antihypertensives and beta-blockers) were frequently used in fallers. It may be necessary to consider alternative medications, modify dosages, or monitor side effects to reduce the risk of falls in older adults who are taking such medications.

When we examined the subclasses of FRIDs, we found that only beta-blockers increased the likelihood of falls. This could be explained by the fact that this drug class can increase the risk of orthostatic hypotension and imbalances leading to falls. In our study, selective and nonselective beta-blockers were more frequently prescribed than other FRID classes, possibly influencing our findings. Nonselective β-blockers could be related to fall risk because of their broader spectrum of action and their potentially negative effect on skeletal muscle. Conflicting results regarding the effects of beta-blockers on falls have been previously published [21, 62–64], and deprescribing FRIDs in isolation has not been shown to reduce falls in older adults in previous meta-analyses [65]. This may be due to the variability among patients and the multifactorial nature of falls [66–68]. Our data suggest that exploring and intervening on a broad range of risk factors including the personalization of pharmacologic treatment is likely necessary to help reduce falls in older adults.

This study had many strengths. Overall, it has a robust methodology, with a detailed statistical analysis that allows the identification of significant predictors of falls among Lebanese community-dwelling older adults. The findings provide valuable information for healthcare professionals in identifying risk factors for falls and implementing customized interventions tailored to individual needs to reduce the risk of falls. Furthermore, the study examined the occurrence of falls within the three months prior to the medication review. While this timeframe may restrict comparisons to international studies, as a duration of 12 months is commonly used, it can mitigate the risk of bias due to memory loss. We included several factors that have not been studied in the literature that may influence the risk of falls, such as behavioral factors. There are several well-studied approaches to fall prevention that either target a single risk factor or focus on multiple risk factors [25–27] but none examine patient behaviors towards their medical treatment. Although patient behaviors were not significantly associated with fall risk in multivariate analysis, this was the first article to address how patients manage their medications in terms of their provision, preparation, or follow-up. In addition, this study included all Lebanese regions, which improves the external validity of our findings. The study is a foundation for further research and will prove essential to the implementation of various plans to control the risk of falls among Lebanese older adults.

The limitations of the study include the questionnaire that didn’t take into consideration all potential risk factors of falls, such as environmental hazards, injuries associated with falls, and the number of previous falls. In addition, it was not possible to correlate the information obtained from the patients with the physician’s medical records due to the challenges of accessing such records in the Lebanese health system. The one-time medication review may not accurately capture the cause-effect relationship between drugs and falls, making it difficult to determine with certainty whether the falls happened before or while using the medication. The list of medications used to identify FRIDs may not be complete and uniform and may not highlight differences between pharmacological subclasses. The study did not consider testing for interactions like the combined effect of disease and medical treatment and excluded participants taking less than 5 drugs which may lead to selection bias.

## Conclusion

In conclusion, this study emphasizes the importance of addressing various risk factors for falls in Lebanese older adults, including loss of appetite, dependency, drowsiness, and specific medications such as beta-blockers. Customized interventions may help prevent falls and improve overall health outcomes in addition to performing continuous medication reviews and analysis of medical prescriptions. Finally, the identification of these factors allows the recognition of the groups that are most susceptible to the occurrence of this outcome and consequently offers important support for the elaboration and planning of Lebanese government policies, actions, and strategies to address this serious public health problem.

## Data Availability

All relevant data are within the paper, its supporting information file, and on the repository Zenodo at the following 10.5281/zenodo.6321171.

## Abbreviations

ADE: Adverse drug events
FRID: Fall risk increasing drugs
PIDP: Potentially inappropriate drug prescribing
MGPIDP-L: Management of potentially inappropriate drug prescribing in Lebanon
OR: Odds Ratio
ATC: Anatomical Therapeutic Chemical
IRB: Institutional Review Board
CI: Confidence Interval
P: Power
